# Evaluation of 50 Ω driver circuits for digital signals based on oscilloscope measurements

**DOI:** 10.1016/j.heliyon.2021.e07674

**Published:** 2021-07-28

**Authors:** Tilman Küpper

**Affiliations:** Hochschule München University of Applied Sciences, Germany

**Keywords:** Electronic engineering, Interface electronics, Line driver, Pulse generator, Trigger output

## Abstract

In this article, various 50 Ω driver circuits for digital signals are compared and evaluated. These circuits can drive different logic families, even over longer cables, with fast rise and fall times of a few nanoseconds. A fully assembled test board and all measurement results are presented. The design files, source codes and measurement results can be freely downloaded from Mendeley Data. Does it make a difference if a solderless breadboard is used instead of a printed circuit board with solid ground plane? Apart from considerable noise on the breadboard's supply rail, the overall signal quality is better than one might expect.

The test board is not only useful for evaluating driver circuits but can also be used directly as a simple pulse generator. Some of the driver circuits presented here show faster rise and fall times than many commercial function generators.

## 1 Introduction

In our mechatronics lab, test equipment for use in teaching and projects is constantly being developed and built. In this context, we decided to upgrade a signal generator with an additional digital trigger output for connecting external components and devices.

The trigger output should be able to drive different logic families, even over longer coaxial cables (RG58) or striplines ([3], p. 646), with fast rise and fall times of a few nanoseconds. However, only a single 5 V supply voltage is available within the signal generator. Typical output stages, such as those found in function generators, cannot be implemented in this way. They usually require supply voltages of more than 5 V and often also negative supply voltages.

The high-level output voltage of 5 V (no load) combined with an output impedance of 50 Ω makes the trigger output quite flexible to use ([8], Figure 17):

• 5 V logic (CMOS or TTL) with high impedance inputs can be connected directly.
• 5 V TTL with 50 Ω inputs can also be connected. The 50 Ω input impedance causes the high-level voltage to drop to 2.5 V, but this is still well above the 5 V TTL threshold of 2.0 V.
• A high-level voltage of 2.5 V is also suitable for driving 3.3 V LVTTL (threshold of 2.0 V) or 2.5 V CMOS (threshold of 1.7 V). In these cases, either 50 Ω inputs are required or feed-through terminators must be connected to the inputs to prevent overvoltage.

Designing a trigger output is certainly not rocket science. It is therefore surprising that one finds only a few suitable circuit proposals in literature and on the internet. In most electronics textbooks this topic is not even discussed [2, 3]. In many projects found on the internet, for example PWM generators or other digital signal generators, the outputs of logic gates, operational amplifiers or microcontrollers are simply led directly to the outside. Of course, this does not lead to defined output impedances, not to mention short-circuit protection.

Horowitz and Hill in “The Art of Electronics” [1] recommend the use of MOSFET gate drivers or HCMOS outputs connected in parallel. An internet search for “50 Ω TTL line driver” also yields some results. Besides the already mentioned MOSFET gate drivers and parallel HCMOS outputs, the use of Single Gate CMOS Logic is recommended [14]. However, one can also find discussions in forums [15, 16, 17, 18], where it becomes clear that there is no “standard solution” for the task discussed here. So, the idea was born to build and test some of these circuit proposals, and to document them in this article:

• All driver circuits are fully documented with schematic, printed circuit board (PCB) layout and measurement results. They can be easily evaluated, compared, and used as building blocks for new projects.
• The test board presented here is not only useful for developing driver circuits but can also be used directly as a simple pulse generator.
• Some of the driver circuits on the test board have faster rise and fall times than many commercial function generators.
• Such a pulse generator can be used, for example, in time domain reflectometry (TDR) experiments.

## 2 Materials and methods

Suggestions for the development of suitable driver circuits can be found, as mentioned, in internet forums or newsgroups. However, these are less reliable sources. The circuit suggestions in “The Art of Electronics” [1] are also not fully documented; neither the printed circuit board (PCB) layout is known nor the type or the technical data of the oscilloscope used.

This is the reason for actually building, systematically analyzing and comparing the following five different driver circuits that can be used to realize digital trigger outputs or 50 Ω line drivers:

1. Parallel microcontroller port pins
2. MCP14A0602 MOSFET gate driver
3. Discrete push-pull output
4. Parallel 74LVC1G04 logic gates
5. Parallel CD74AC04M logic gates

After a brief description of the principle of operation, the PCB layout is shown for each circuit. Rise and fall times are recorded with an oscilloscope, with no load (open output) and with a 50 Ω load resistor. The oscilloscope used is a PicoScope 3206A with an analog bandwidth of 200 MHz [7].

A brief discussion follows as to whether the requirements in Table 1 can be met.

**Table 1:** Requirements for the trigger output.

High-level output voltage (no load)	5 V
Low-level output voltage	0 V
Output impedance	50 Ω
Single supply voltage	5 V
Rise and fall time	<10 ns
Short-circuit protection	Continuous

**Figure 1:**
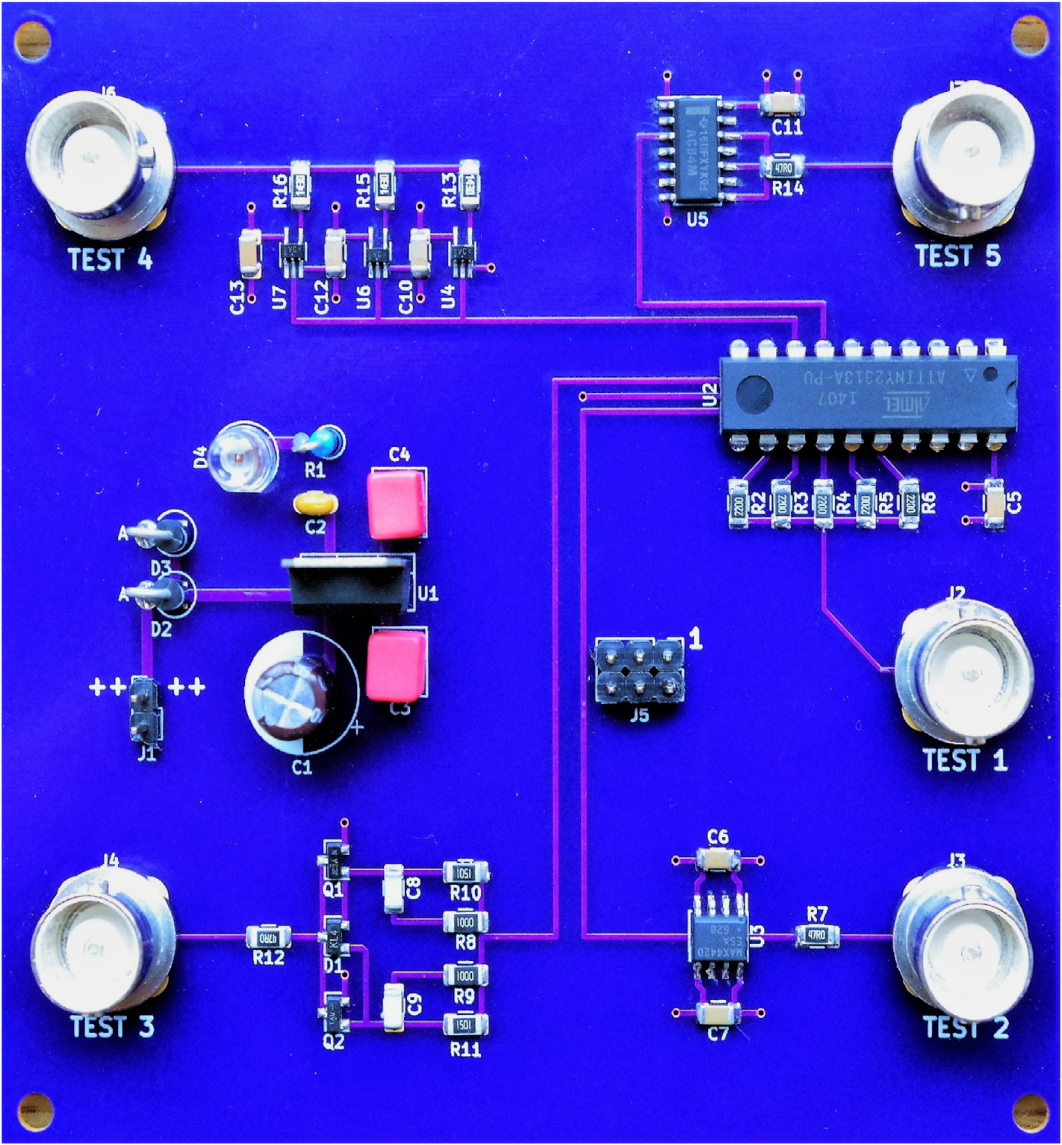
Fully assembled test PCB.

The driver circuits are built and tested on a four-layer PCB with solid ground plane and VCC layer. The layer stackup is: L1 - signals, L2 - GND, L3 - VCC, L4 - signals. Figure 1 shows the fully assembled test board. A linear voltage regulator provides the 5 V supply voltage for the individual driver circuits and for an ATtiny2313 microcontroller. This microcontroller generates test signals for the five separate outputs. The board layout was designed with KiCAD. All design files, bill of materials, source codes and measurement results can be freely downloaded from Mendeley Data [5, 6], see Table 2.

• testboard.zip: KiCAD files with schematic and PCB layout
• firmware.zip: ATtiny2313 firmware, generating test signals (Atmel Studio 7)
• oscope.zip: Oscilloscope measurements of all driver circuits, with and without load resistor
• simul3.zip: LTspice simulation of the discrete push-pull driver circuit
• bill_of_materials.pdf: List of components on the test board

**Table 2:** Design files and oscilloscope data.

File name	File type	License	Location
testboard.zip	KiCAD files	CC BY 4.0	https://doi.org/10.17632/zj4fxws38x.2
firmware.zip	Firmware	CC BY 4.0	https://doi.org/10.17632/zj4fxws38x.2
oscope.zip	Oscilloscope data	CC BY 4.0	https://doi.org/10.17632/zj4fxws38x.2
simul3.zip	LTspice simulation	CC BY 4.0	https://doi.org/10.17632/zj4fxws38x.2
bill_of_materials.pdf	PDF document	CC BY 4.0	https://doi.org/10.17632/zj4fxws38x.2

The intention is to create a solid starting point for subsequent work, which does not yet exist in this form.

Does it make a difference if a solderless breadboard is used instead of a PCB? To answer this question, a variant of the fourth driver circuit (parallel CD74AC04 logic gates) is built on a solderless breadboard and analyzed with the oscilloscope, too (section 5).

## 3 Driver circuits and measurement results

### 3.1 Parallel microcontroller port pins

**Figure 2:**
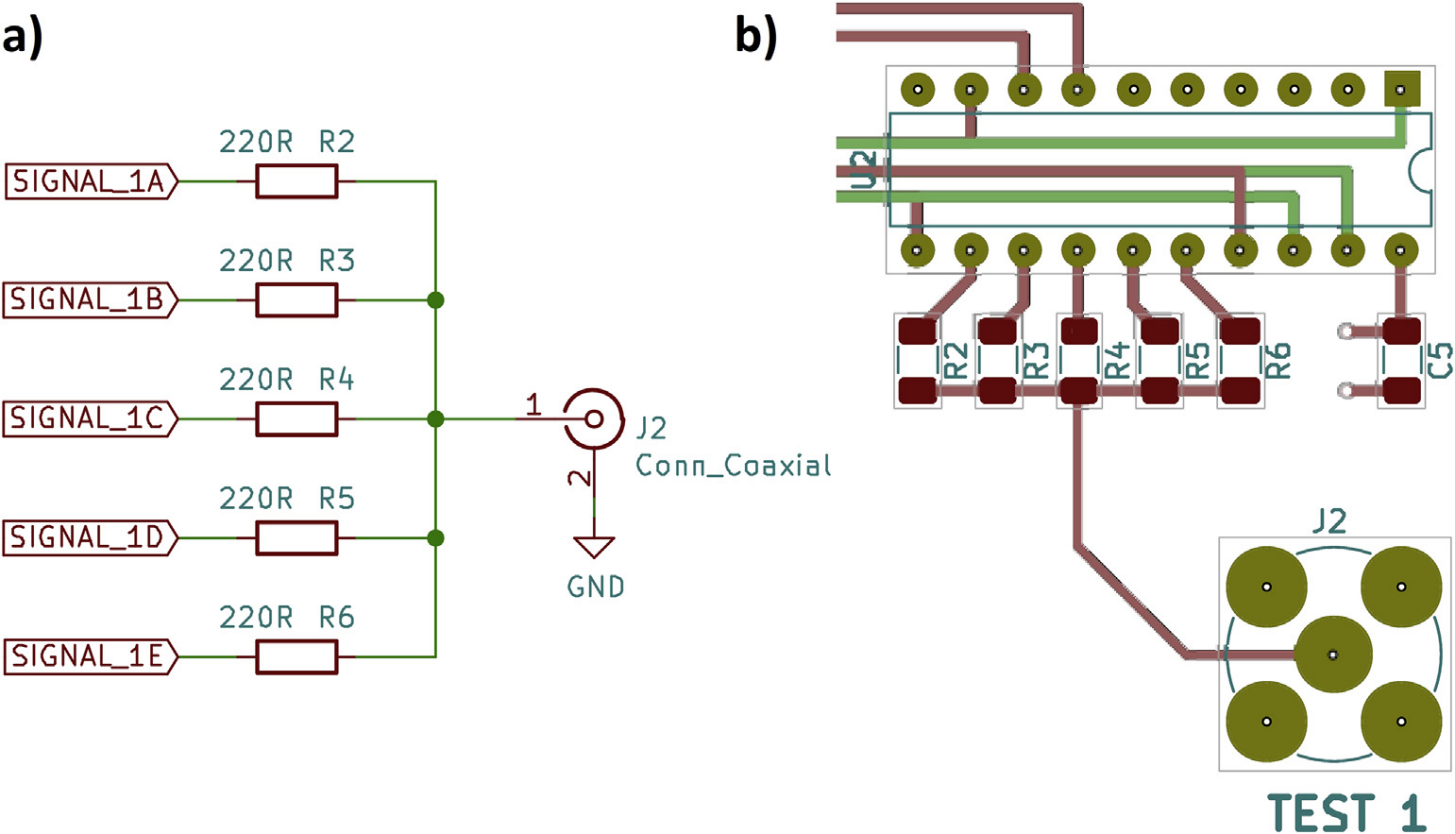
Parallel port pins, a) schematic, b) PCB layout.

The first driver circuit is not a “circuit” at all, only five microcontroller port pins with resistors connected in parallel. On the one hand, the resistors lead to an output impedance of 50 Ω, and on the other hand, the required short-circuit protection. Figure 2 shows the schematic and the PCB layout.

**Figure 3:**
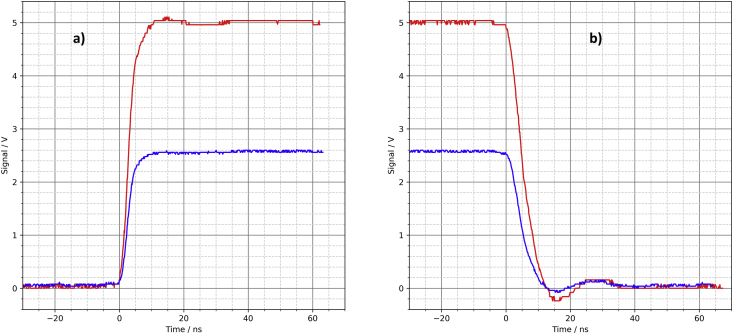
Parallel port pins, oscilloscope, a) rising edge, b) falling edge.

On the oscilloscope (Figure 3) you can see clean edges with rise times of approx. 6 ns and fall times of approx. 9 ns. There are slight overshoots on the falling signal edges. The blue signals were recorded with a 50 Ω load resistor, the red signals with no load (open output). This applies to all figures, also in the following sections.

A big advantage of this first driver circuit is that there is no relevant propagation delay from the microcontroller port pins to the driver output. A disadvantage is that many (five) pins are needed. The microcontroller firmware must also take care to always switch all pins simultaneously.

If a load resistor of 50 Ω is connected, a current of 100 mA is delivered from the trigger output (and thus also from the microcontroller port pins). For the ATtiny2313 microcontroller used here, this is quite close to the absolute maximum ratings specified in the datasheet ([11], p. 198). Consequently, only one trigger output per microcontroller can be realized in this way. Otherwise, short circuits at the trigger output could damage the microcontroller. This is another disadvantage of this circuit variant.

### 3.2 MCP14A0602 MOSFET gate driver

Horowitz and Hill recommend in “The Art of Electronics” the use of TC4421 MOSFET gate drivers ([1], p. 861, Driving Cables: Far-end termination). They also repeat this suggestion in electronics forums on the internet [18]. A 47 Ω resistor at the output of such a gate driver results in the desired trigger output impedance of 50 Ω. Gate drivers are robust integrated circuits; they can deliver currents of several amperes and have internal protection circuits against overload and overtemperature.

**Figure 4:**
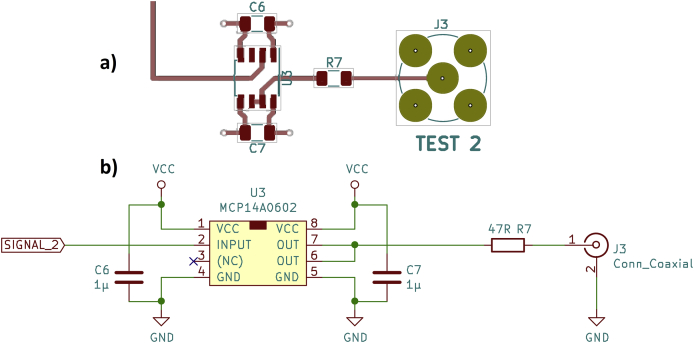
MCP14A0602 MOSFET gate driver, a) PCB layout, b) schematic.

**Figure 5:**
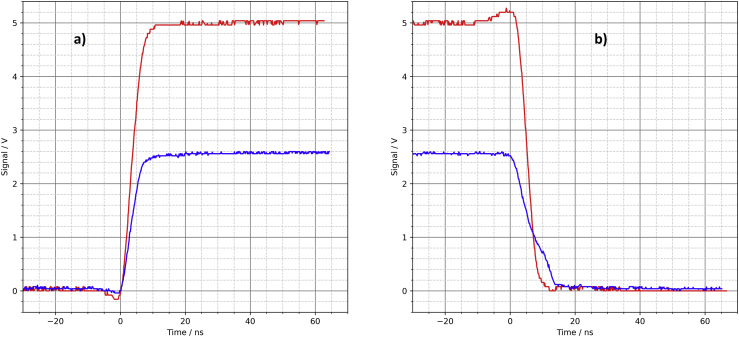
MCP14A0602 MOSFET gate driver, oscilloscope, a) rising edge, b) falling edge.

An MCP14A0602 MOSFET gate driver is used on the test board (see Figure 4). Its rise and fall times ([12], Figures 2-2 and 2-4) are slightly faster compared to the type proposed by Horowitz and Hill ([13], Figures 2-2 and 2-5).

With no load (open output), the oscilloscope (Figure 5) shows clean edges with rise and fall times of approx. 6 ns. However, with a 50 Ω load resistor, the fall time increases to approx. 11 ns. If this moderately fast fall time is acceptable for the intended application, such a MOSFET gate driver is a very robust circuit option. Due to the additional 47 Ω resistor at the output of the gate driver, the trigger output should be practically indestructible.

A major disadvantage of this second driver circuit is the high propagation delay of the MOSFET gate driver of approx. 35 ns (see [5, 6], file oscope.zip, for measurement data). This applies to both the rising and the falling edges. The MCP14A0602 datasheet ([12], p. 1) specifies typical propagation delays of “only” 22 ns, which, however, requires a supply voltage of at least 12 V.

### 3.3 Discrete push-pull output

**Figure 6:**
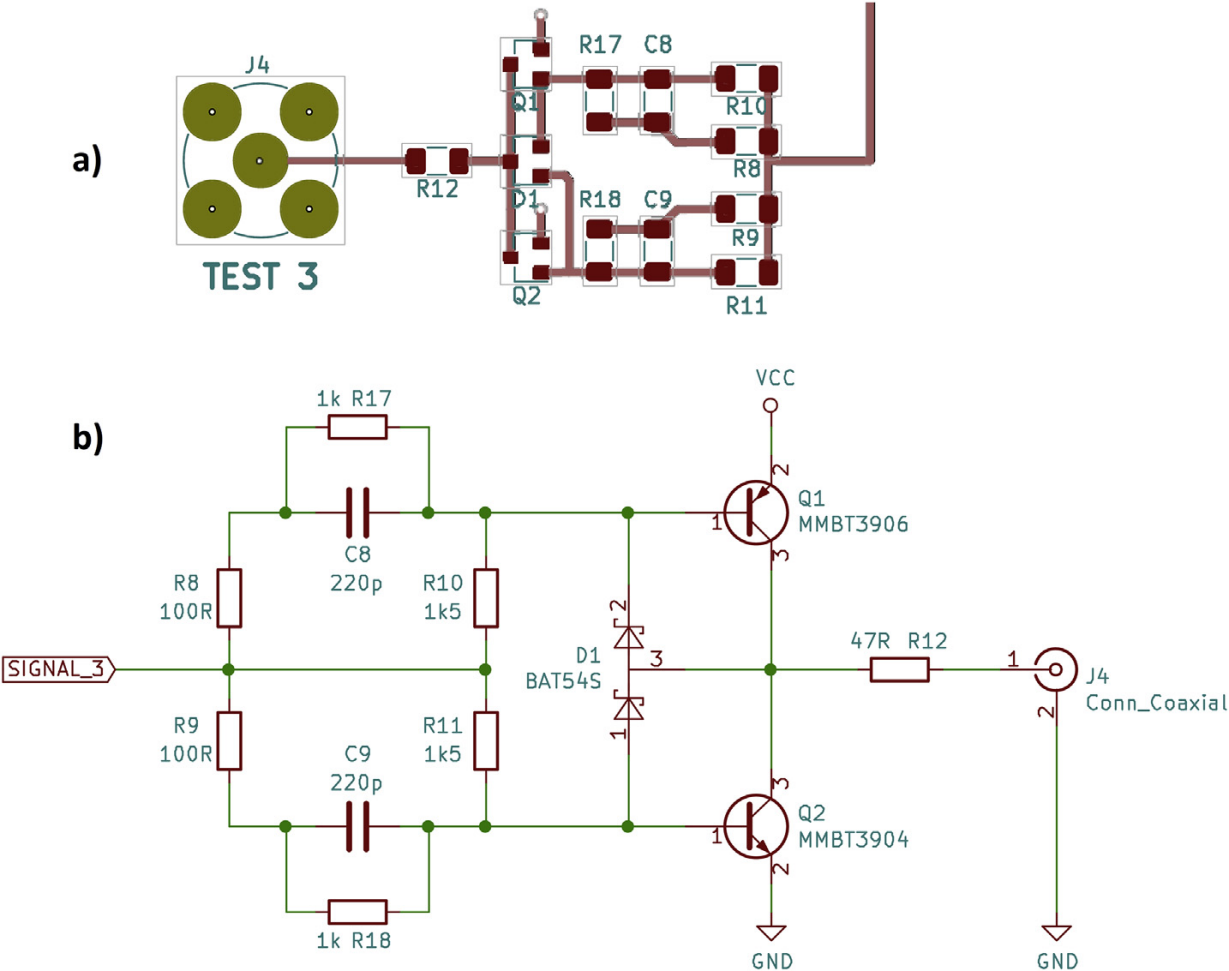
Discrete push-pull output, a) PCB layout, b) schematic.

The third driver circuit does not need any integrated circuits but transistors, resistors, and capacitors. It is a discrete push-pull output (Figure 6). In addition to the transistors MMBT3906 (PNP) and MMBT3904 (NPN), there are speed-up capacitors (C8, C9) and Baker clamps (D1) to accelerate the switching process (see [2], p. 227 and [4], p. 16). One advantage of this circuit is that it can be built with standard components only.

R17 and R18 are (only) needed for frequencies above approx. 100 kHz. Without these resistors there is not enough time to discharge the capacitors between two switching edges, causing ugly “shark fins” on the output signal.

**Figure 7:**
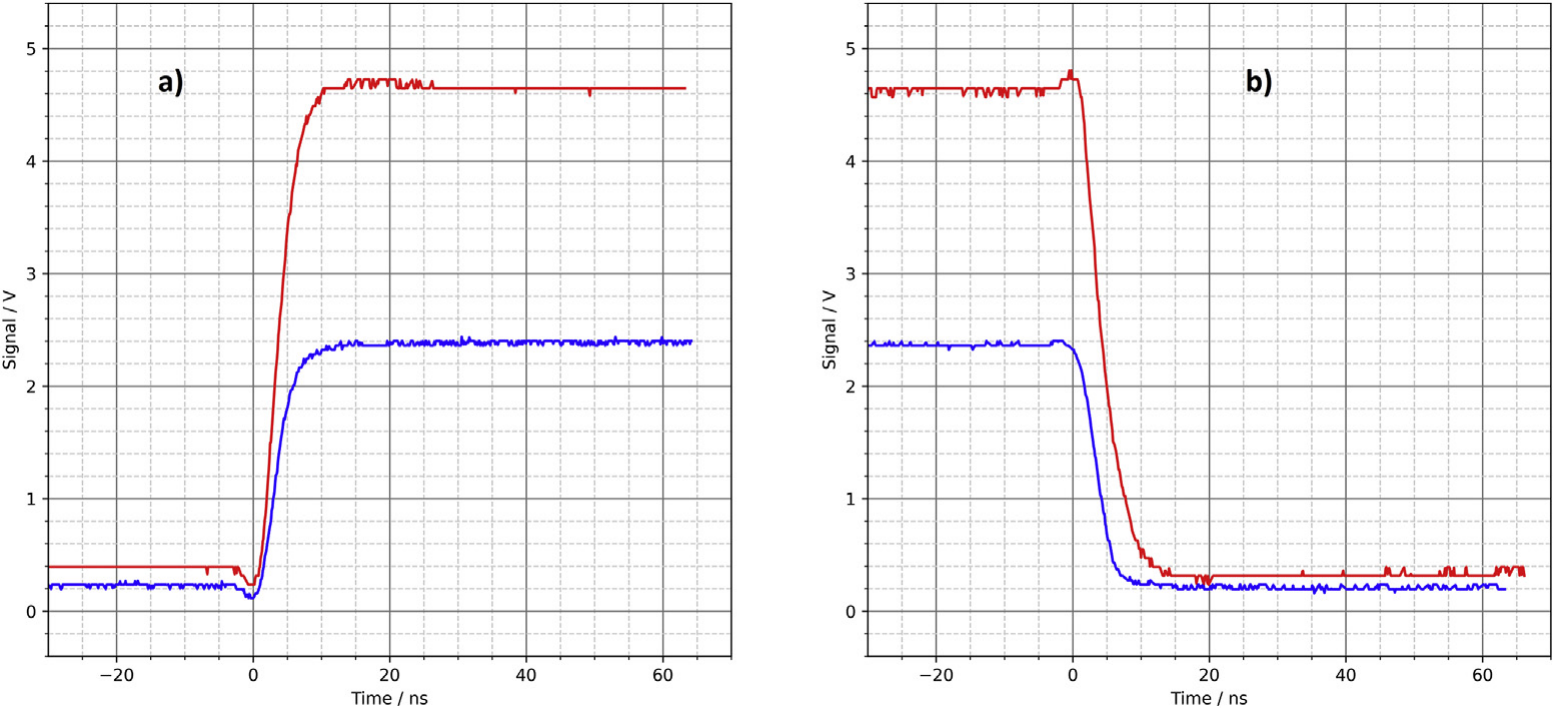
Discrete push-pull output, oscilloscope, a) rising edge, b) falling edge.

The oscilloscope shows rise and fall times of 5–7 ns (Figure 7). However, the collector-emitter saturation voltage of a few 100 mV results in neither the desired high-level voltage of 5 V (or 2.5 V with 50 Ω load resistor) nor the desired low-level voltage of 0 V being reached. This circuit is probably not useable as a universal driver circuit.

### 3.4 Parallel 74LVC1G04 logic gates

**Figure 8:**
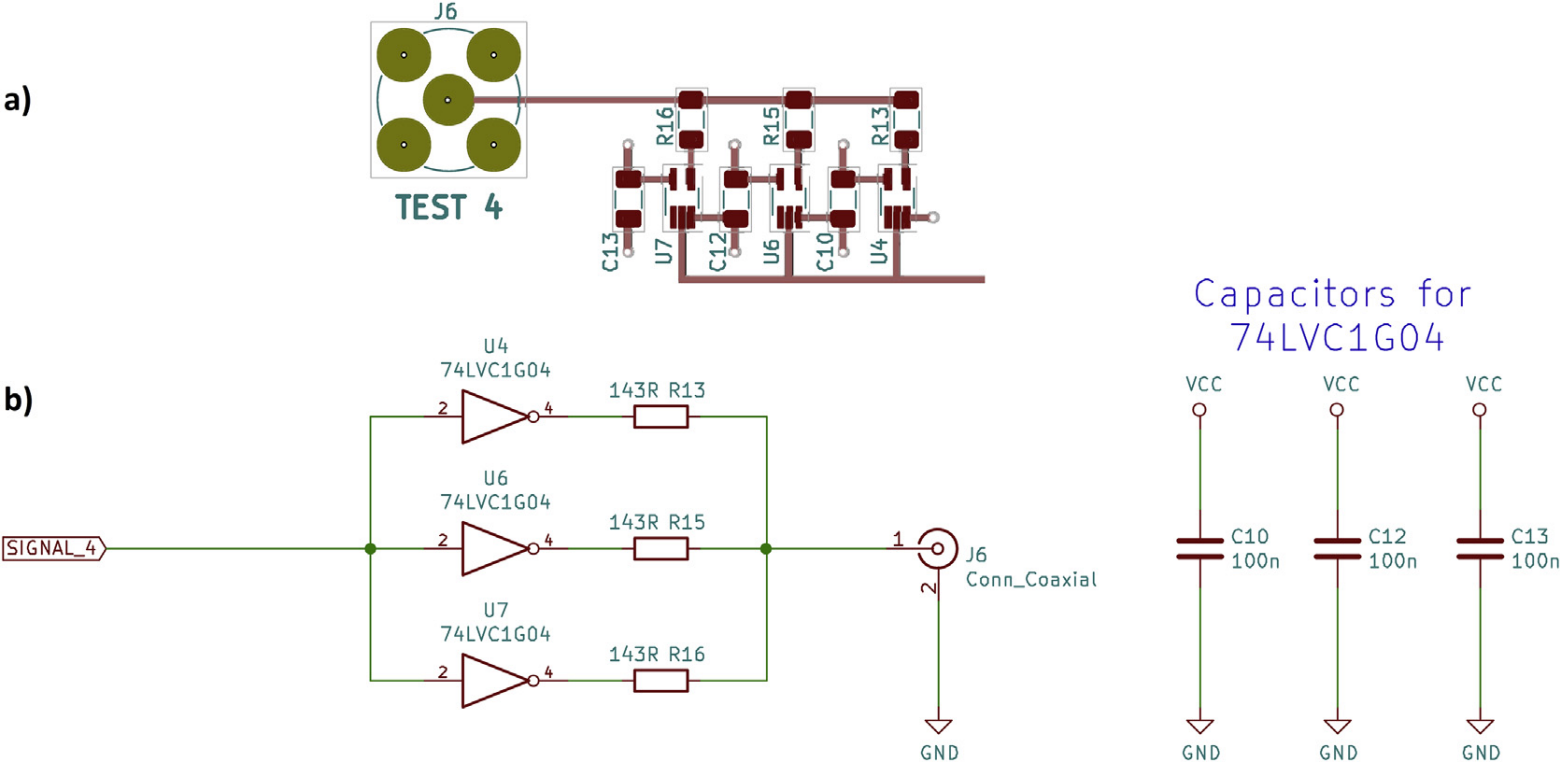
Parallel 74LVC1G04 logic gates, a) PCB layout, b) schematic.

The fourth driver circuit was found on the internet. On June 14, 2018, Tom Gardner wrote in the newsgroup sci.electronics.design: “If you drive a 50 Ω cable with three parallel (74lvc1g∗ + 143 Ω in series) then the risetime is <<1 ns (probably <300 ps, but I can't measure that).” [14] That's reason enough to include this driver circuit on the test board as well (see Figure 8).

**Figure 9:**
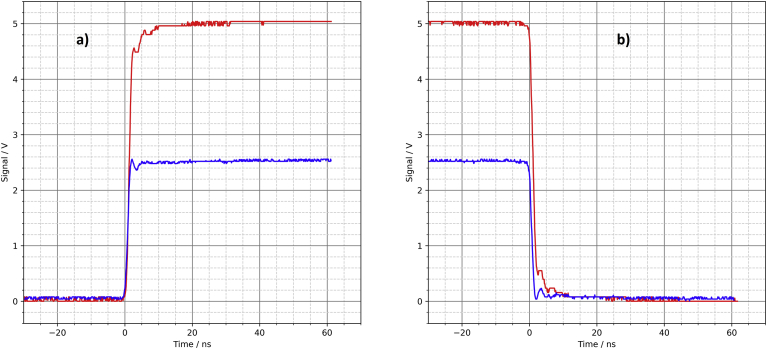
Parallel 74LVC1G04 logic gates, oscilloscope, a) rising edge, b) falling edge.

The oscilloscope shows rapid rise and fall times of approx. 1.5 ns (Figure 9). Only very small oscillations at the end of the switching edges are visible. At this point, it should be noted that the rise time of the oscilloscope is specified by the oscilloscope manufacturer as 1.75 ns [7]. Consequently, the actual rise and fall times of the driver circuit are likely to be much faster, well below 1 ns. With no load (open output) it is noticeable, both for the rising edge and the falling edge, that the last half volt before reaching the final voltage takes a little more time (approx. 5 ns). However, this should not be a problem in practical applications of this driver circuit.

With a 50 Ω load resistor at the output of this driver circuit, each logic gate must supply a current of 16.7 mA. With the output shorted, the current increases to 33.3 mA per gate. These values are well below the absolute maximum ratings specified in the datasheet ([10], limiting value: 50 mA, recommended value up to 32 mA with a supply voltage of 4.5 V). As with the first driver circuit in section 2.1, the three resistors provide both an output impedance of 50 Ω as well as the required short-circuit protection.

### 3.5 Parallel CD74AC04M logic gates

**Figure 10:**
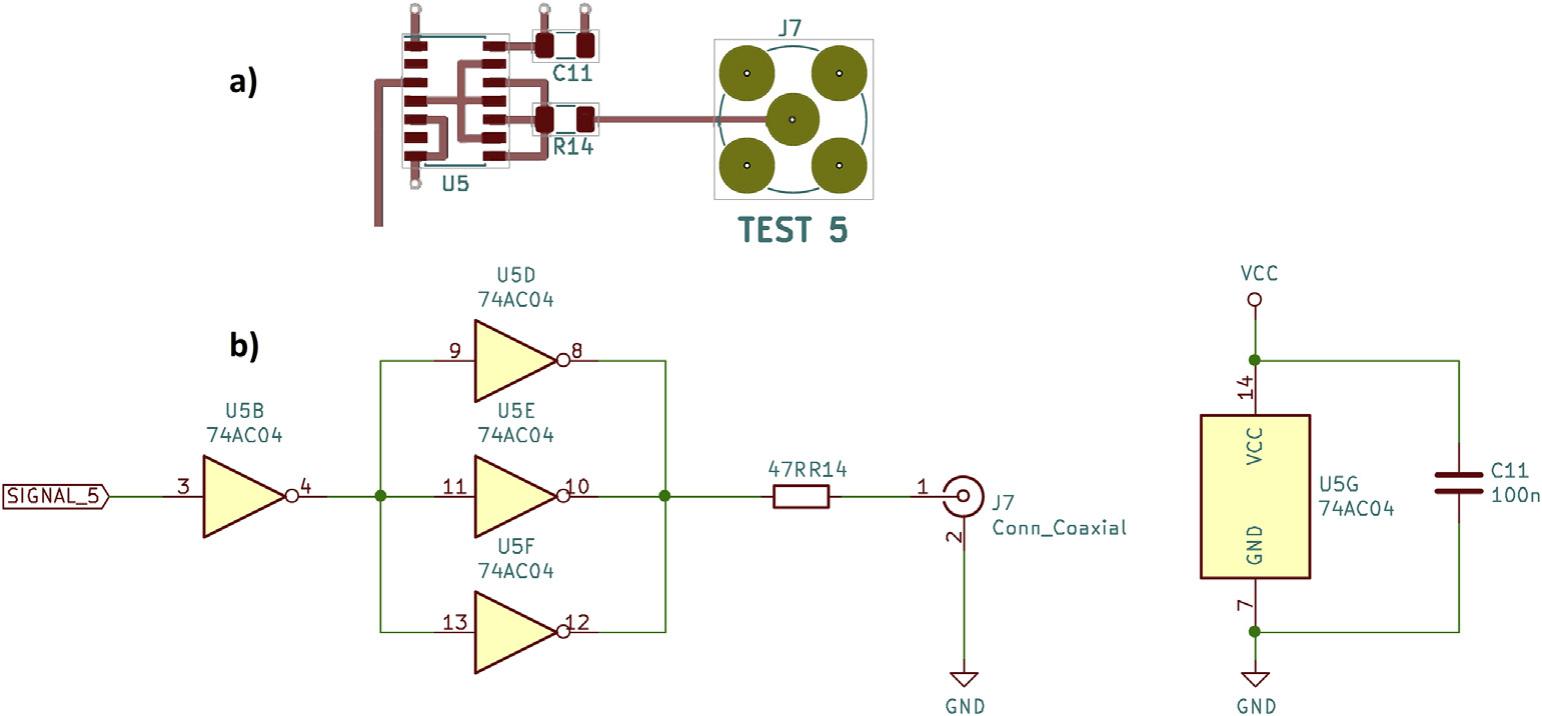
Parallel CD74AC04M logic gates, a) PCB layout, b) schematic.

The fifth and last driver circuit on the test board is again based on a recommendation by Horowitz and Hill in “The Art of Electronics” ([1], p. 861, Driving Cables: Far-end termination). Similar suggestions can be found in [19] and [20]. Three parallel CD74AC04M logic gates are connected to the output via a 47 Ω resistor. This is the same idea as the fourth driver circuit. Here, however, only a single integrated circuit is required, which contains all the logic gates used (see Figure 10).

**Figure 11:**
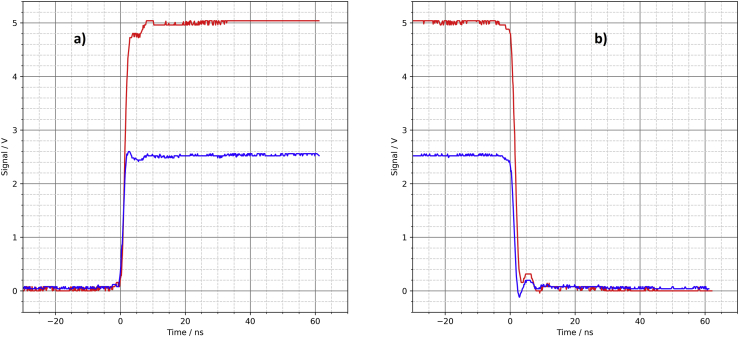
Parallel CD74AC04M logic gates, oscilloscope, a) rising edge, b) falling edge.

The signals captured with the oscilloscope are similar in shape and speed to those of the fourth driver circuit with rise and fall times of approx. 2 ns (Figure 11). This is again at the limit of what can be captured with the oscilloscope used here.

The CD74AC04M datasheet ([9], p. 2) states a max. continuous output current of ±50 mA and a max. continuous current through VCC or GND of ±100 mA. Accordingly, the required short-circuit protection is given, although the total package current rating is only just met.

## 4 Conclusions

The requirements for a flexible, fast digital trigger output as listed in Table 1 are best met by the fourth (parallel 74LVC1G04 logic gates) and fifth (parallel CD74AC04M logic gates) driver circuits. The particularly simple first driver circuit as well as the robust second driver circuit may also be useful in special cases but show significantly slower rise and fall times.

Another aspect that might be important when choosing a driver circuit is the propagation delay from the input to the output of the circuit. The propagation delays for all five driver circuits are listed in Table 3; they were determined with 50 Ω load resistors ([5, 6], file oscope.zip). Regarding the propagation delays, the fourth and fifth drivers can again be recommended:

**Table 3:** Propagation delays.

No.	Driver circuit	Rising edge	Falling edge
1	Parallel microcontroller ports	<1 ns	<1 ns
2	MCP14A0602 MOSFET gate driver	36 ns	35 ns
3	Discrete push-pull output	2 ns	2 ns
4	Parallel 74LVC1G04 logic gates	<1 ns	2 ns
5	Parallel CD74AC04M logic gates	5 ns	6 ns

The high propagation delay of the MOSFET gate driver (second driver circuit) is striking. This alone will significantly complicate the use of this circuit in many applications.

## 5 Solderless breadboards

Finally, to answer the last question that was asked at the beginning of this article, does it make a difference if a solderless breadboard is used instead of a PCB with a solid ground plane?

Solderless breadboards show capacitive coupling between the individual contact strips (up to approx. 20 pF between the power supply rails) and quite high point contact resistance. In addition, there are flying leads and significantly longer legs of components (compared to SMD components on PCBs). Especially for high frequency applications these breadboards have a bad reputation.

**Figure 12:**
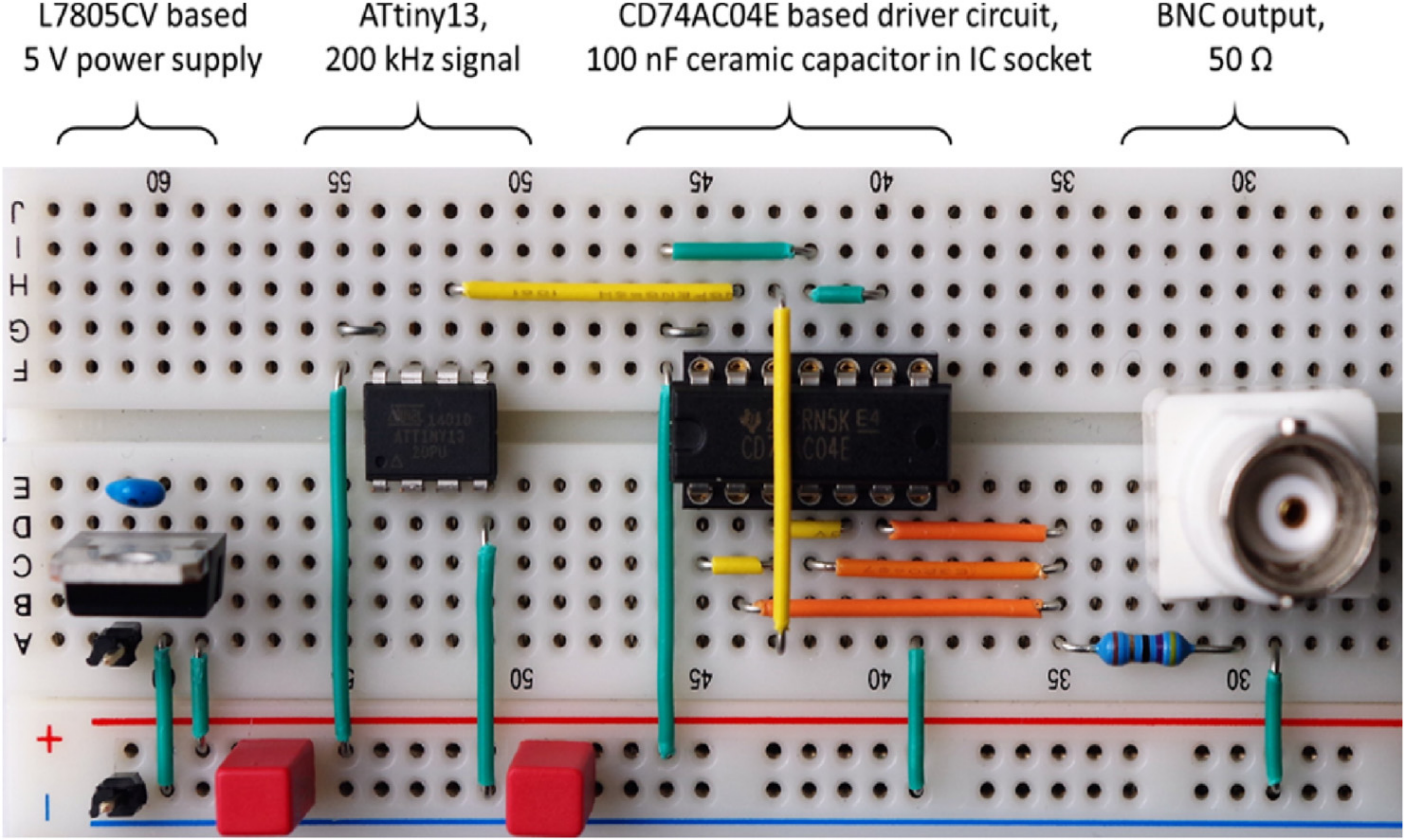
Solderless breadboard, parallel CD74AC04E logic gates.

Figure 12 shows the fifth driver circuit on a solderless breadboard. On the same board are an L7805CV-based power supply and an ATtiny13 microcontroller that generates a 200 kHz test signal.

**Figure 13:**
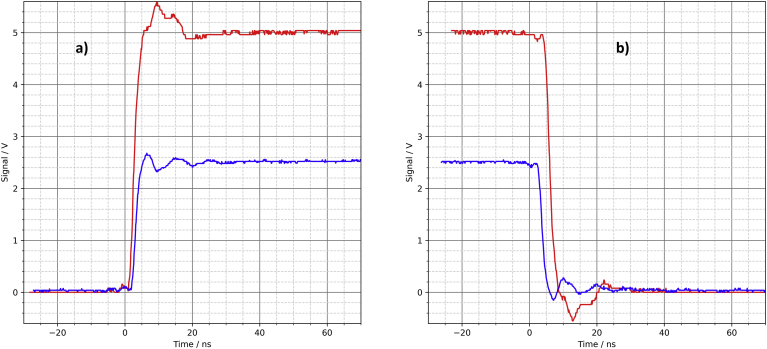
Solderless breadboard, oscilloscope, a) rising edge, b) falling edge.

**Figure 14:**
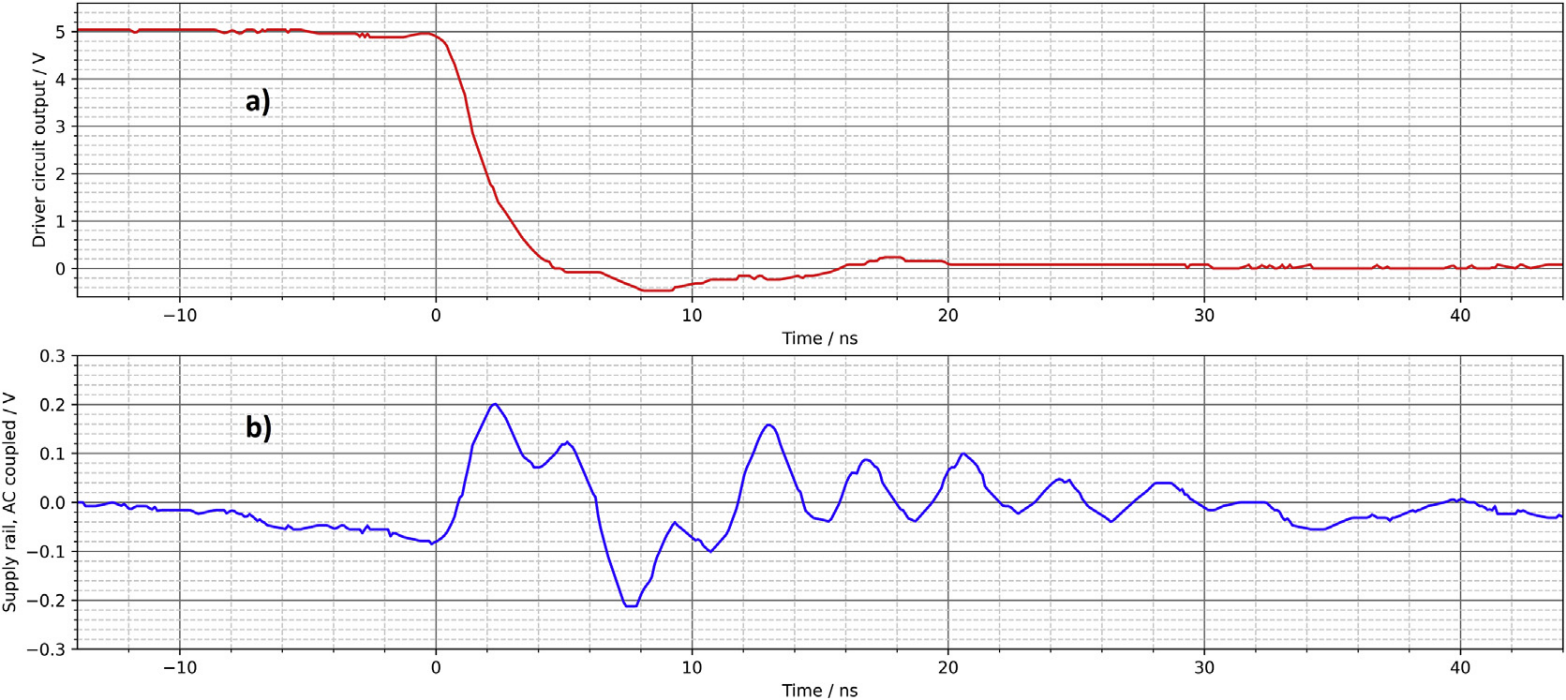
Solderless breadboard, a) output of the driver circuit, b) noise on the supply rail.

The signal at the output of the driver circuit does not look too bad (Figure 13). The overshoot at the switching edges is slightly larger compared to the PCB. The solderless breadboard also shows significant noise on the supply rail (Figure 14). If there are other (analog) components on the same breadboard, this can lead to interference. However, the overall signal quality is better than the author expected.

## Declarations

### Author contribution statement

Tilman Küpper: Conceived and designed the experiments; Performed the experiments; Analyzed and interpreted the data; Wrote the paper.

### Funding statement

This work was supported by Hochschule München, QualiFIVE (99361/9936516).

### Data availability statement

Data associated with this study has been deposited at Mendeley under the accession number https://doi.org/10.17632/zj4fxws38x.2.

### Declaration of interests statement

The authors declare no conflict of interest.

### Additional information

No additional information is available for this paper.
